# Brachial artery transposition versus catheters as tertiary vascular access for maintenance hemodialysis: a single-center retrospective study

**DOI:** 10.1038/s41598-021-03860-1

**Published:** 2022-01-10

**Authors:** Yu Soma, Masaaki Murakami, Eiji Nakatani, Yoko Sato, Satoshi Tanaka, Kiyoshi Mori, Akira Sugawara

**Affiliations:** 1 Department of Nephrology, Shizuoka General Hospital, 4-27-1 Kitaando, Aoi-ku, Shizuoka, 420-8527 Japan; 2 Division of Clinical Biostatistics, Research Support Center, Shizuoka General Hospital, Shizuoka, 420-8527 Japan; 3 Graduate School of Public Health, Shizuoka Graduate University of Public Health, 4-27-2 Kitaando, Aoi-ku, Shizuoka, 420-0881 Japan

**Keywords:** Renal replacement therapy, Haemodialysis

## Abstract

Some hemodialysis patients are not suitable for creation of an arteriovenous fistula (AVF) or arteriovenous graft (AVG). However, they can receive a tunneled cuffed central venous catheter (tcCVC), but this carries risks of infection and mortality. We aimed to evaluate the safety and effectiveness of brachial artery transposition (BAT) versus those of tcCVC. This retrospective study evaluated hemodialysis patients who underwent BAT or tcCVC placement because of severe heart failure, hand ischemia, central venous stenosis or occlusion, inadequate vessels for creating standard arteriovenous access, or limited life expectancy. The primary outcome was whole access circuit patency. Thirty-eight patients who underwent BAT and 25 who underwent tcCVC placement were included. One-year patency rates for the whole access circuit were 84.6% and 44.9% in the BAT and tcCVC groups, respectively. The BAT group was more likely to maintain patency (unadjusted hazard ratio: 0.17, 95% confidence interval: 0.05–0.60, *p* = 0.006). The two groups did not have significantly different overall survival (log-rank *p* = 0.146), although severe complications were less common in the BAT group (3% vs. 28%, *p* = 0.005). Relative to tcCVC placement, BAT is safe and effective with acceptable patency in hemodialysis patients not suitable for AVF or AVG creation.

## Introduction

The United States, Japan, and Europe have aging populations of patients with end-stage renal disease^1–3^. Their comorbidities and/or blood vessel conditions can make these patients unsuitable for creation of an arteriovenous fistula (AVF) or arteriovenous graft (AVG) for hemodialysis (HD). Furthermore, cardiovascular disease, including congestive heart failure (CHF), is highly prevalent and the primary cause of death among HD patients^4–8^. Moreover, a retrospective analysis revealed that creating an AVF or an AVG was associated with right ventricular (RV) dilation, which was independently associated with worsening of CHF and an increased risk of death among HD patients^9^. Vascular access (VA)-induced ischemia is also common among patients who have peripheral circulatory disorders, such as older patients, patients with diabetes, and patients who undergo frequent operations for arteriovenous (AV) access^10,11^.

The European Society for Vascular Surgery guidelines indicate that long-term placement of a tunneled cuffed central venous catheter (tcCVC) may be appropriate for HD patients with VA-induced ischemia, CHF, or a limited life expectancy^12^. The Kidney Disease Outcomes Quality Initiative guidelines also indicated that a tcCVC can be used in cases where VA is difficult because of outflow vein problems, such as central venous occlusion or stenosis^13^. However, some observational studies have identified a poorer survival rate among HD patients who received a tcCVC than among patients who underwent creation of an AVF or AVG^14–18^. Moreover, placement of a CVC is associated with an increased risk of infection^19–21^.

Some reports have described using an artery as an alternative VA strategy^22,23^, and brachial artery transposition (BAT) is a recognized alternative to tcCVC placement in patients who are not suitable for AVF or AVG creation^1,24,25^. The BAT strategy for VA uses the brachial artery for blood delivery and the superficial veins for blood return. Therefore, if either the superficial brachial artery or superficial vein has a problem and cannot function as VA or be dialyzed, the entire access circuit will be lost.

Moreover, cannulation of the brachial artery must be delayed for > 2 weeks until the brachial artery has completely adhered to the connective tissue. Finally, although several reports have described BAT, few studies have compared the outcomes to those from tcCVC as a more standard option. Therefore, this study evaluated the safety and effectiveness of BAT and tcCVC as tertiary access strategies in HD patients who were not suitable for AVF or AVG creation.

## Results

### Patient characteristics

**Figure 1:**
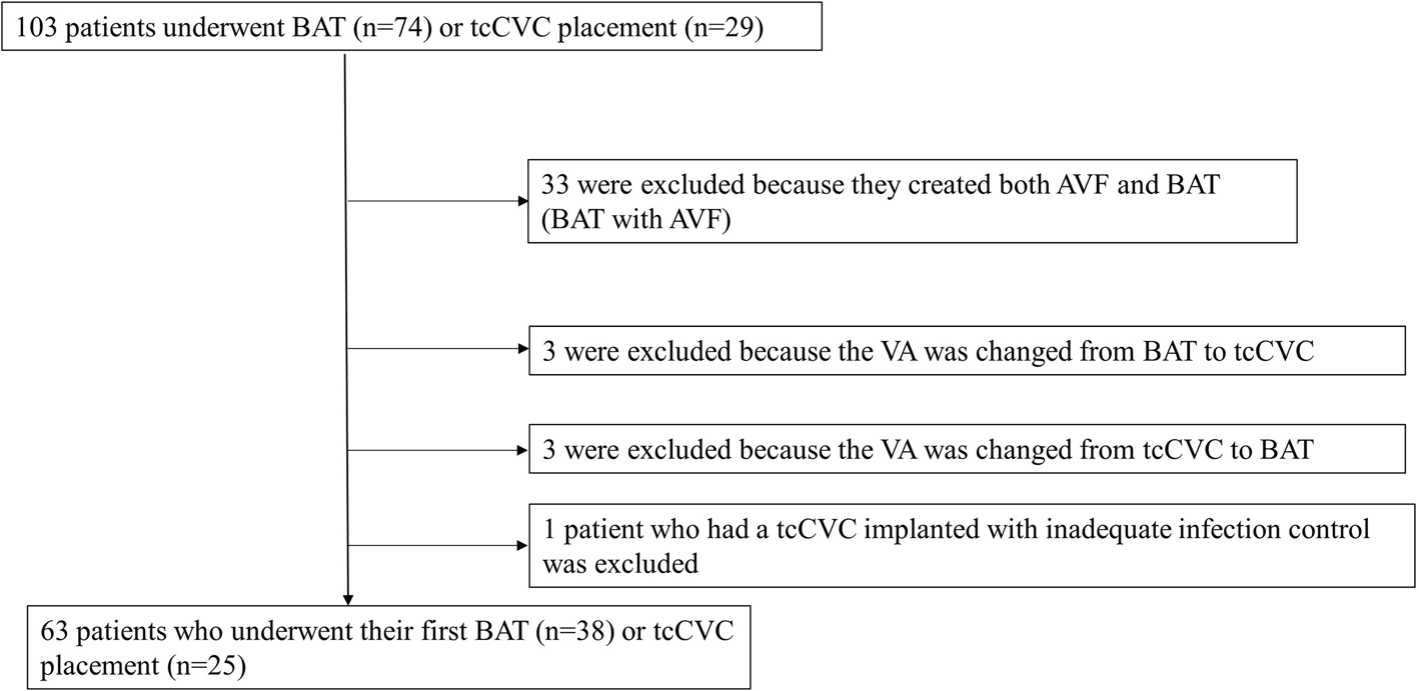
The study flowchart. Among patients who underwent BAT (n = 74) or tcCVC placement (n = 29), we excluded patients who created both AVF and BAT, had previously undergone BAT (n = 3) or tcCVC placement (n = 3), as well as 1 patient who had a tcCVC implanted with inadequate infection control. *AVF* arteriovenous fistula, *BAT* brachial artery transposition, *tcCVC* tunneled cuffed central venous catheter, *VA* vascular access.

One hundred and three patients underwent surgery for BAT or tcCVC. Thirty-three patients had an AVF attached to the BAT and were excluded because an AVF could be created in these patients and the indications were not met. In these patients, the length of the AVF was less than 10 cm, and the BAT was created to increase the area available for puncture. Patients who changed VA from BAT to tcCVC (n = 3) and tcCVC to BAT (n = 3) were excluded because of the possible influence of the reciprocal relationship. Patients with tcCVC who underwent surgery with poorly controlled infections were excluded (n = 1). This study finally included 38 patients who underwent BAT and 25 patients who underwent permanent tcCVC placement for HD during the study period (Fig. 1). The baseline characteristics were generally similar, although the BAT group had a higher proportion of patients with heart failure with reduced ejection fraction (HFrEF) (Table 1). The detailed causes of CHF and hand ischemia are shown in Supplementary Table 1.

**Table 1:** Characteristics of patients who underwent brachial artery transposition or tunneled cuffed central venous catheter placement.

Variable	Category or unit	BAT (n = 38)	tcCVC (n = 25)	*p* Value
Age	1 year	72.45 ± 8.95	75.2 ± 14.72	0.359
Sex	Male	24 (63.2)	12 (48.0)	0.301
BMI	1 kg/m^2^	19.81 ± 3.14	20.34 ± 3.04	0.505
Diabetes	Present	15 (39.5)	10 (40.0)	> 0.999
Hypertension	Present	30 (81.1)	20 (80.0)	> 0.999
IHD	Present	18 (47.4)	8 (32.0)	0.298
PAD	Present	9 (23.7)	21 (84.0)	0.538
Stroke	Present	10 (26.3)	7 (28.0)	> 0.999
HFrEF	Present	23 (60.5)	8 (32.0)	0.039
HVD	Present	9 (23.7)	3 (12.0)	0.334
COPD	Present	7 (18.4)	2 (8.0)	0.176
Cancer	Present	4 (10.5)	6 (24.0)	0.298
Antiplatelet drugs	Present	19 (50.0)	7 (28.0)	0.117
Warfarin	Present	8 (21.1)	4 (16.0)	0.752
Causes of ESRD	Diabetes	11 (28.9)	8 (32.0)	0.912
	BNS	10 (26.3)	6 (24.0)	
	CGN	7 (18.4)	3 (12.0)	
	Others	10 (26.3)	8 (32.0)	
Indication	CHF *	13 (34.2)	8 (32.0)	0.749
	Hand ischemia*	10 (26.3)	5 (20.0)	
	CVS/O	5 (13.2)	2 (8.0)	
	Inadequate vessels	8 (21.1)	9 (36.0)	
	Limited life expectancy^†^	2 (5.3)	1 (4.0)	
Original type of VA	None	16 (42.1)	11 (44.0)	0.462
	AVF	14 (36.8)	6 (24.0)	
	AVG	8 (21.1)	8 (32.0)	
Dialysis history	1 year	7.50 (0.00–27.00)	3.00 (0.00–21.00)	0.180

Data are reported as number (%), mean ± standard error, or median (range).

*BAT* brachial artery transposition, *tcCVC* tunneled cuffed central venous catheter, *BMI* body mass index, *IHD* ischemic heart disease, *PAD* peripheral artery disease, *HFrEF* heart failure with reduced ejection fraction, *HVD* heart valve disease, *COPD* chronic obstructive pulmonary disease, *BNS* benign nephrosclerosis, *CGN* chronic glomerulonephritis, *CHF* chronic heart failure, *CVS/O* central venous stenosis or occlusion, *VA* vascular access.

*Details regarding CHF and hand ischemia are provided in Supplementary Table 1.

^†^In the BAT group, 1 patient selected BAT because of stage 4 rectal cancer and another patient selected BAT because of stage 4 lung adenocarcinoma. In the tcCVC group, 1 patient selected tcCVC placement because of a stage 4 intraventricular tumor.

### Dialysis progress

The dialysis progress is shown in Table 2. The mean follow-up intervals were 537 ± 435 days for the BAT group and 196 ± 291 days for the tcCVC group. In the BAT group, the brachial artery was cannulated after approximately 3 weeks if wound healing was observed, which involved the use of a 17-gauge needle on the blood delivery side and puncture of any superficial vein using a 16–17-gauge needle on the blood return side. The mean interval from the BAT operation to its first successful use for dialysis was 28.9 ± 14.5 days. The proportions of cases with adequate dialysis (Kt/V of > 1.2) were 93.3% in the BAT group and 78.9% in the tcCVC group, although the difference was not significant (*p* = 0.19).

**Table 2:** Dialysis progress in the brachial artery transposition and tunneled cuffed central venous catheter groups throughout follow-up.

Dialysis progress	BAT (n = 38)	tcCVC (n = 25)	p Value
Time from BAT procedure to first successful use of dialysis (days)	23 (13–76)	NA	NA
17-gauge needle for BAT	34 (100)*	NA	NA
QB^†^	200 (140–250) (n = 34)	180 (120–250) (n = 25)	0.153
Kt/V^‡^	1.43 (1.12–1.84) (n = 30)	1.40 (0.72–1.86) (n = 19)	0.485
Kt/V > 1.2	28/30 (93.3)	15/19 (78.9)	0.190
Kt/V > 1.4	18/30 (60.0)	10/19 (52.6)	0.768

Data are reported as number (%) or median (range).

*BAT* brachial artery transposition, *tcCVC* tunneled cuffed central venous catheter, *QB* blood pump flow delivered to the dialyzer, *Kt/V* dialysis rate.

*In the BAT group, the BAT was not used for 4 patients, which was not related to death before the first cannulation (n = 2), death before initiating dialysis for chronic renal failure (n = 1), and conservative treatment without initiation of dialysis (n = 1).

^†^QB was recorded for all patients who underwent dialysis (n = 34).

^‡^Kt/V was measured for 30 patients in the BAT group and 19 patients in the tcCVC group.

### Patency rates for the whole access circuit

**Figure 2:**
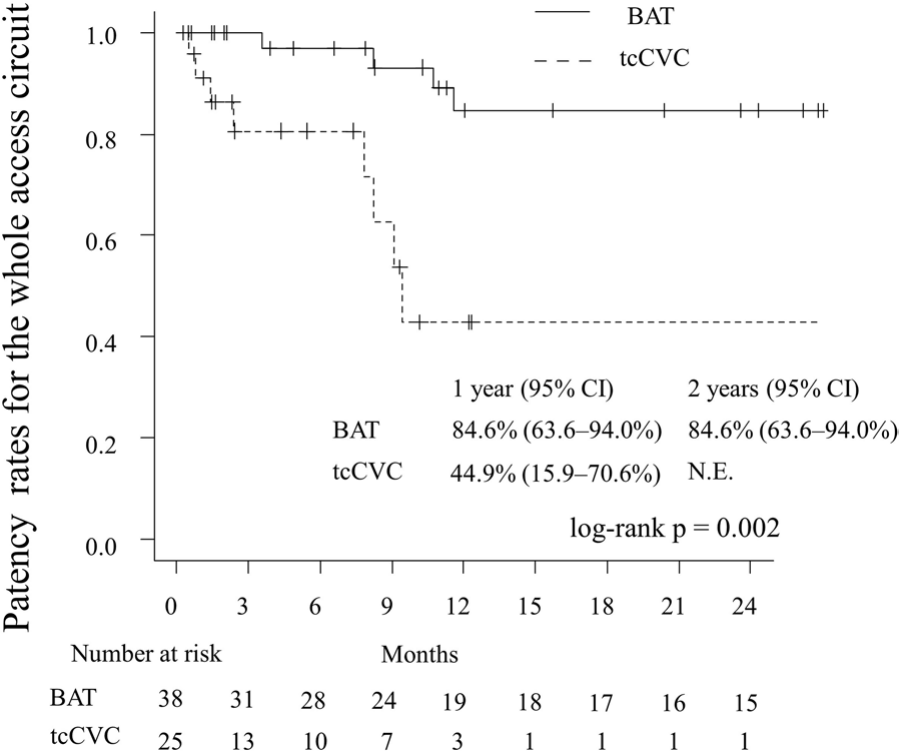
Patency rates for the whole access circuit throughout the follow-up period. The 1-year patency rates for the whole access circuit were 84.6% for the BAT group and 44.9% for the tcCVC group. *BAT* brachial artery transposition, *tcCVC* tunneled cuffed central venous catheter.

In the BAT group, the surgical success rate was 94.7%, as 2 patients died because of VA-unrelated acute coronary syndrome before the first successful cannulation. During the observation period, the BAT group had a primary patency rate of 92.9% and a secondary patency rate of 100%. The 1-year patency rates for the whole access circuit were 84.6% in the BAT group and 44.9% in the tcCVC group (*p* = 0.002) (Fig. 2). Univariable Cox proportional hazard analyses revealed that the BAT group had a greater likelihood of whole access circuit patency (HR: 0.17, 95% CI 0.05–0.60, *p* = 0.006, Table 3). Furthermore, the BAT group had a higher patency rate in the analyses that were adjusted for age, hypertension, HFrEF, and antiplatelet drugs (all *p* = 0.022 to 0.002, Table 4).

**Table 3:** Univariate Cox regression analysis of whole access circuit patency.

Variable (reference)	Category or unit	HR (95% CI)	*p* Value
Age, years	1	0.98 (0.94–1.04)	0.530
Sex (male)	Female	1.70 (0.52–5.58)	0.383
BMI, kg/m^2^	1	0.99 (0.80–1.22)	0.915
Diabetes (absent)	Present	0.30 (0.07–1.41)	0.129
Hypertension (absent)	Present	0.26 (0.07–0.92)	0.037
IHD (absent)	Present	0.56 (0.15–2.13)	0.397
PAD (absent)	Present	0.99 (0.21–4.57)	0.986
Stroke (absent)	Present	0.26 (0.03–2.01)	0.195
HFrEF (absent)	Present	0.30 (0.08–1.13)	0.075
HVD (absent)	Present	0.83 (0.18–3.84)	0.801
COPD (absent)	Present	< 0.001 (0.00–Inf)	0.998
Cancer (absent)	Present	2.10 (0.56–7.94)	0.273
Antiplatelet drugs (absent)	Present	0.12 (0.02–0.96)	0.046
Warfarin (absent)	Present	0.84 (0.18–3.90)	0.825
Causes of ESRD (diabetes)	BNS	1.71 (0.29–10.26)	0.556
	CGN	1.58 (0.22–11.23)	0.650
	Others	2.56 (0.47–14.04)	0.279
Indication (CHF)	Hand ischemia	1.00 (0.17–6.01)	0.947
	CVS/O	< 0.001 (0.00–Inf)	0.998
	Inadequate vessels	3.05 (0.67–13.81)	0.149
	Limited life expectancy	4.71 (0.78–28.51)	0.092
Original type of VA (none)	AVF	0.53 (0.10–2.92)	0.468
	AVG	1.62 (0.43–6.08)	0.473
Dialysis history, years	1	0.99 (0.91–1.07)	0.728
Kind of VA (tcCVC)	BAT	0.17 (0.05–0.60)	0.006

*HR* hazard ratio, *CI* confidence interval, *Inf* infinity, *BMI* body mass index, *IHD* ischemic heart disease, *PAD* peripheral artery disease, *HFrEF* heart failure with reduced ejection fraction, *HVD* heart valve disease, *COPD* chronic obstructive pulmonary disease, *BNS* benign nephrosclerosis, *CGN* chronic glomerulonephritis, *CHF* chronic heart failure, *CVS/O* central venous stenosis or occlusion, *VA* vascular access, *BAT* brachial artery transposition, *tcCVC* tunneled cuffed central venous catheter.

**Table 4:** Effectiveness of brachial artery transposition and tunneled cuffed central venous catheter in terms of whole access circuit patency.

Adjusted variable (ref)	Category or unit	HR (95% CI)	P Value
None	–	0.17 (0.05–0.60)	0.006
Age	1 year	0.17 (0.05–0.59)	0.005
Sex (male)	Female	0.18 (0.05–0.64)	0.008
Hypertension (absent)	Present	0.12 (0.03–0.83)	0.002
HFrEF (absent)	Present	0.21 (0.06–0.77)	0.018
Antiplatelet drugs (absent)	Present	0.23 (0.06–0.81)	0.022

*HR* hazard ratio, *CI* confidence interval, *HFrEF* heart failure with reduced ejection fraction.

In the BAT group, loss of whole access circuit patency was observed in 3 patients, which was related to loss of the return side veins in all 3 cases. There were no cases of hand ischemia requiring amputation due to occlusion or ischemia of the brachial artery. In the tcCVC group, loss of whole access circuit patency was observed in 8 patients, which was related to catheter dysfunction (2 patients) or catheter-related bloodstream infection (6 patients). Information regarding the new or additional VA strategies is provided in Supplementary Table 2.

### Overall survival rates

**Figure 3:**
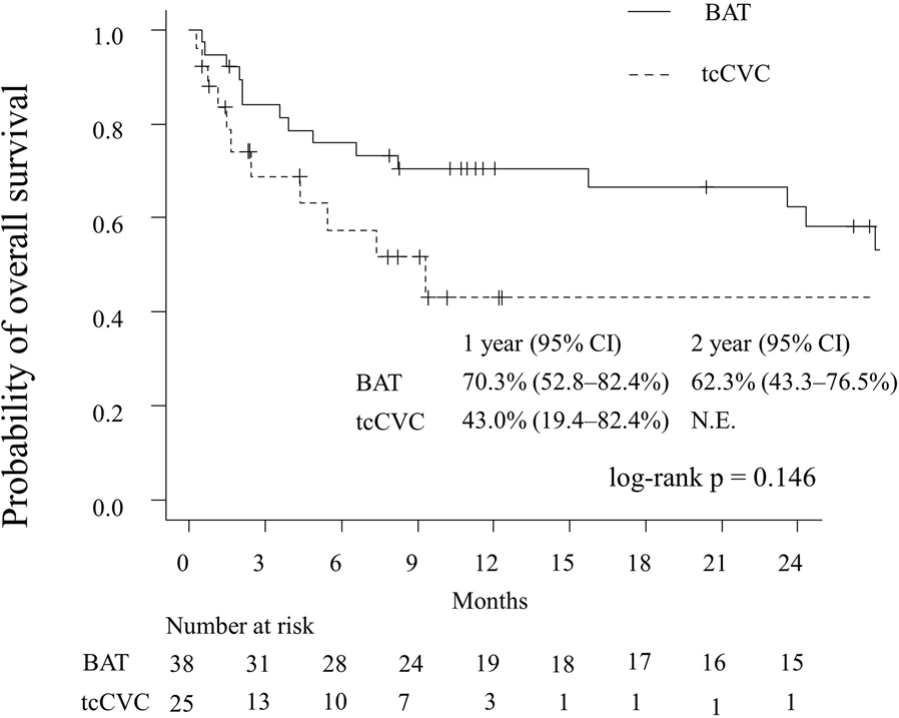
Overall survival rates throughout the follow-up period. The 1-year overall survival rates were 70.3% for the BAT group and 43.0% for the tcCVC group. *BAT* brachial artery transposition, *tcCVC* tunneled cuffed central venous catheter.

During the observation period, 19 patients died in the BAT group and 13 patients died in the tcCVC group. The causes of death are described in Supplementary Appendix 1. The 1-year overall survival rates were 70.3% in the BAT group and 43.0% in the tcCVC group. No significant differences were observed in the Kaplan–Meier analysis (*p* = 0.146, Fig. 3) or the unadjusted Cox regression analysis (Supplementary Table 3).

### Complications

The complications are listed in Table 5. The BAT group had a significantly lower rate of severe complications (3% vs. 28%, *p* = 0.005). In addition, the BAT group was significantly less likely to develop VA-related infection (0% vs. 24%, *p* = 0.003). Only 1 patient in the BAT group experienced a severe complication, which involved a CTCAE grade 3 brachial artery aneurysm, and secondary patency of the BAT was restored after surgical resection of the brachial artery aneurysm. All other complications in the BAT group were CTCAE grade ≤ 2 and did not require surgery, with patency of the whole access circuit preserved throughout the observation period. No patients died because of BAT-related complications.

**Table 5:** Complications in the brachial artery transposition and tunneled cuffed central venous catheter groups during follow-up.

Complication	BAT, mean: 537 days (n = 38)	tcCVC, mean: 196 days (n = 25)	*p* Value
	n (%)	n (%)	
Delayed wound healing*	2 (5.3)	NA	NA
Edema delaying cannulation*	1 (2.6)	NA	NA
Hematoma*	1 (2.6)	NA	NA
Lymphorrhea*	1 (2.6)	NA	NA
Brachial artery aneurysm	1 (2.6)	NA	NA
Thrombosis	0 (0)	5 (20.0)^†^	0.008
Infections caused by vascular access	0 (0)	6 (24.0)	0.003
Grade ≥ 3 CTCAE complications^‡^	1 (2.6)	7 (28.0)	0.005

*BAT* brachial artery transposition, *tcCVC* tunneled cuffed central venous catheter, *CTCAE* common terminology criteria for adverse events.

*In the BAT group, delayed wound healing was CTCAE grade 1 or grade 2 (1 case each), edema delaying cannulation was grade 1 (1 case), hematoma was grade 1 (1 case), and lymphorrhea was grade 2 (1 case).

^†^In the tcCVC group, 2 patients developed thrombosis that was managed by adjusting the catheter’s position or using anticoagulants (heparin, urokinase), which preserved the patency of the whole access circuit. The other 3 patients required catheter exchange and lost patency of the whole access circuit.

^‡^In the BAT group, 1 patient developed a CTCAE grade 3 brachial artery aneurysm. In the tcCVC group, 6 patients developed catheter-related bloodstream infections (CRBSIs) and 2 of these patients developed pulmonary embolism (PE) and superior vena cava syndrome (SVCS) as complications of thrombosis. One patient had CTCAE grade 3 CRBSI and PE/SVCS, while the other 6 patients had CTCAE grade 3 complications.

In the tcCVC group, 6 patients experienced VA-related catheter-related bloodstream infection, which were generally CTCAE grade 3, and 5 patients experienced catheter-related thrombosis. One patient developed superior vena cava syndrome and pulmonary embolism, and ultimately underwent open heart surgery because of CTCAE grade 4 thrombosis in the superior vena cava. No patients died because of tcCVC-related complications.

## Discussion

Previous studies have indicated that the catheter-related bloodstream infection is several times more common than AVF and AVG, and 12-month rate of catheter-related bloodstream infection is approximately 50% among patients who receive HD^26,27^, and infection is the second most common cause of hospitalization and death among HD patients^28,29^. Furthermore, the incidence of catheter-related infection can reach 0.28–1.7 episodes per 1000 CVC days^30–32^. Thus, strategies are needed to reduce the rate of VA-induced infection. The frequency of catheter infections may be affected by its definition. The definition of CRBSI in our study was based on the KDOQI-2019 guideline. The present study revealed an incidence of 1.28 episodes per 1000 CVC days in the tcCVC group, which is comparable to the previously reported rates, although the BAT group had a lower risk of VA-induced infection. Moreover, the BAT group had a significantly lower rate of serious complications.

Venous access through superficial veins may be difficult in the early stages due to unstable puncture sites, although the puncture site is stabilized subsequently, and venous puncture becomes easier in many cases. If the puncture site is unstable, a return blood route cannot be obtained, and the whole BAT access circuit is abandoned. In the present study, the whole BAT access circuit was abandoned within 1 year and, therefore, loss of the whole BAT access circuit beyond 1 year could not be observed. Therefore, although the BAT circuit required more time to prepare (vs. tcCVC placement), it may be a useful option for patients who have a life expectancy of > 1 year, given the lower likelihood of severe complications and longer patency of the whole access circuit. Furthermore, we observed that the BAT group had relatively good primary and secondary patency rates throughout follow-up. This may be because, in cases with AVG or AVF creation, the major causes of lost patency are stenosis of the graft-vein anastomosis and thrombotic occlusion due to intimal hyperplasia plus impaired remodeling (related to reduced blood flow) at the juxta-anastomotic stenosis^33–36^. In contrast, the BAT may provide relatively physiological hemodynamics and a lower shear stress (vs. AVF and AVG), which may prolong the patency of the circuit. In previous reports, primary patency at 1 year of BAT has been as high as over 90%^24,37^. In these studies, although about 2–4% of patients developed brachial artery embolism, the majority of patients were asymptomatic, and there were no cases of serious hand ischemia such as upper limb amputation. When BAT was created, side branches were ligated generally. The formation of collateral blood vessels may have improved the tolerability of brachial artery thrombosis. Therefore, BAT is considered to be a relatively safe VA to use, even considering the risk of thromboembolism.

Among patients who undergo dialysis 3 times per week, the recommended Kt/V target is 1.4 and the lower limit is 1.2^38^. A prospective observational study has also indicated that the rates of inadequate dialysis (Kt/V of < 1.2) were 19.2% among patients with an AVF, 9.9% among patients with an AVG, and 27.5% among patients with a CVC^39^. The present study revealed that only 6.7% of patients in the BAT group had inadequate dialysis, which may be related to the independent inflow and outflow routes in these patients. A nationwide Japanese survey has also indicated that the mean Kt/V values were 1.37 for artery transposition, 1.28 for tcCVC, and 0.97 for temporary CVC^40^. Thus, the Kt/V value in our tcCVC group was relatively good, and it is possible that further accumulation of cases might identify a significantly better Kt/V value for BAT (vs. tcCVC).

Antiplatelet drugs are used to reduce AV access thrombosis, although these drugs do not significantly reduce AV access failure^41,42^. The KDOQI-2019 guideline suggests that the use of systemic anticoagulants for improvement of the patency of CVCs has more disadvantages than advantages^13^. There is also no information regarding whether antiplatelet drugs can prolong CVC patency. The present study revealed that loss of the whole BAT access circuit was only related to loss of the superficial veins as the return side vessels. Therefore, it is unclear whether antiplatelet drugs can prolong patency in this setting.

### Limitations

The present study has several limitations. First, this retrospective single-center study only evaluated patients who were considered eligible for BAT or tcCVC placement, suggesting that the results are not representative of all dialysis patients. Second, we did not consider long-term patency of the whole access circuit, and short-term loss of patency in the BAT group was only related to loss of the return side veins. Thus, we suggest that strategies may be needed to protect the return side vessels and preserve the patency of the whole BAT access circuit. Third, we observed that BAT provided sufficient blood flow (140–250 mL/min), although various required flow rates have been reported in the United States (300 mL/min), the United Kingdom (250 mL/min), Germany (200 mL/min), and Japan (160 mL/min)^43^. A nationwide Japanese survey has indicated that artery transposition can provide a flow rate of > 300 mL/min; however, such cases accounted for approximately 1.5% of the studied population^40^, which suggests that further international studies are needed. Fourth, several observational studies have suggested that the relatively poor prognosis of patients with tcCVC placement (vs. AVF) is related to their poor general condition, which may suggest that physicians prefer CVCs for patients who have a poor general condition^44,45^. The same thing could happen in the selection of BAT and tcCVC. Fifth, we adjusted the analyses for known confounding factors, although unidentified confounding factors (e.g., patient status) might also have contributed to our findings. Finally, it is possible that the tcCVC was preferentially selected if there was a paucity of superficial veins as a return route.

## Conclusion

The present study revealed that, relative to tcCVC placement, BAT had a longer patency period for the whole access circuit and a lower risk of severe complications, especially VA-related infection. Thus, BAT may be a safe and effective tertiary access strategy in HD patients who are not suitable for AVF or AVG creation. However, further studies may be needed to confirm the long-term rates of patency, complications, and overall survival.

## Materials and methods

### Ethics

This study complies with the “Ethical Principles for Medical Research Involving Human Subjects” of the Ministry of Health, Labour and Welfare and the Ministry of Education, Culture, Sports, Science, and Technology. In accordance with these guidelines, the Shizuoka General Hospital Research Ethical Committee determined that individual patient informed consent was not necessary because this study was an analytical study of existing information and patients were given the right to refuse participation through disclosure. With the approval of the committee (SGHIRB#2020089), the disclosure document was made available on the website of Shizuoka General Hospital, and the information of individuals was anonymized for analysis.

### Study design, setting, and patients

We retrospectively identified HD patients who underwent BAT (BAT group) or tcCVC placement (tcCVC group) as a tertiary VA strategy at our local general hospital between January 2016 and September 2020. Patients were included if their original VA strategy involved no VA, AVF creation, or AVG creation. Patients were excluded if their original VA strategy involved BAT or tcCVC placement. In addition, 1 patient was excluded because the tcCVC was implanted with inadequate infection control.

### Data collection

The patients’ medical records were reviewed to collect data regarding age, sex, body mass index, diabetes, hypertension, ischemic heart disease, peripheral artery disease, stroke, HFrEF, left ventricular ejection fraction of < 40%, heart valve disease, chronic obstructive pulmonary disease, cancer, antiplatelet drugs, warfarin, cause of end-stage renal disease, indications for tertiary VA, original type of VA, and dialysis history (in years). Patient outcomes were monitored through visits at our hospital and the last visit was defined as the last follow-up date. In cases where patients underwent dialysis at other hospitals, their physicians were contacted to confirm VA patency, complications, and survival.

### Indications for tertiary vascular access

The indications for BAT or tcCVC placement were severe CHF, hand ischemia, central venous occlusion or stenosis, inadequate vessels for standard AV access, or limited life expectancy. Severe CHF was identified based on the presence of HFrEF, previous/concurrent severe ischemic heart disease, and/or previous/concurrent heart valve disease. Hand ischemia was defined as signs and symptoms of stage ≥ 2 VA-induced ischemia (loss of pain sensation, or pain during HD or exercise). Cases with central venous occlusion or stenosis were identified based on inadequate or delayed contrast enhancement or non-enhancement during digital subtraction angiography, which had been performed to investigate the cause of inadequate dialysis and/or edema caused by VA. Inadequate vessels for standard AV access were identified based on artery and vein diameters of < 2 mm at the planned anastomosis site. Limited life expectancy was defined as expected survival of < 1 year.

### Procedures

All patients in the tcCVC group received a Palindrome Precision catheter (Chronic Dialysis Catheter, Minneapolis, MN, USA).

**Figure 4:**
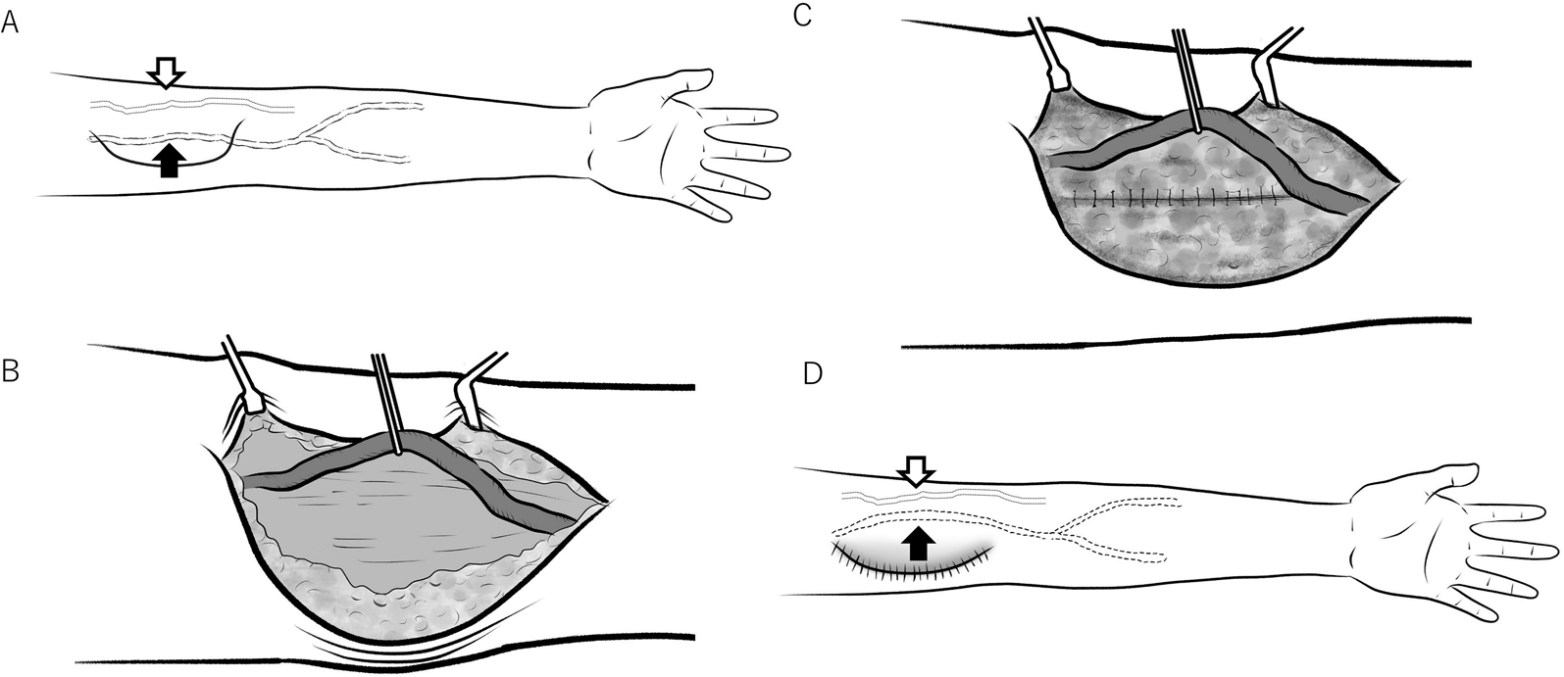
Schema of brachial artery transposition surgical procedure. (**A**) Continuous skin incision was made over the brachial artery from the elbow to the axilla. (**B**) The brachial artery is then liberated entirely from the surrounding subcutaneous tissue. (**C**) A subcutaneous fat pocket was created to hold the brachial artery. The subcutaneous fat was sewn using absorbable 4-0 nylon sutures under the brachial artery. (**D**) After closing the wound, the transposed brachial artery is positioned slightly radially. The black arrows indicate the brachial artery. The white arrows indicate a superficial vein used as a return route. The superficial vein has not been moved.

The schema for the BAT surgery is shown in Fig. 4. Local or conduction anesthesia was used for the BAT group. A continuous 15–20-cm skin incision was made over the brachial artery from the elbow to the axilla. The brachial artery was then liberated entirely from the surrounding subcutaneous tissue, and all brachial artery branches were carefully ligated using 4-0 silk sutures before they were divided. A subcutaneous fat pocket was created to hold the brachial artery, which was relocated from the ulnar side to the upper arm's radial side. The subcutaneous fat was sewn using absorbable 4-0 nylon sutures under the brachial artery to prevent prolapse from the subcutaneous pocket before skin closure.

### Outcomes

The primary outcome was the patency rate of the whole access circuit. In the BAT group, primary patency was calculated from the initial operation to the first re-operation for BAT-related complications, and secondary patency was calculated from the initial operation to the abandonment of BAT (i.e., secondary patency was judged present for all re-operations that restored patency and did not lead to abandonment). The patency of the whole BAT access circuit was calculated from the initial operation to the abandonment of BAT as a functional VA (e.g., because of blood return dysfunction), and patency of the whole circuit was not considered lost until an unsuccessful reoperation. The whole access circuit also includes the transposed brachial artery and the veins for blood return. In the tcCVC group, primary patency was calculated from the initial catheter placement until any intervention that did not involve catheter removal or replacement. Patency of the whole tcCVC access circuit was defined as the interval between catheter placement and catheter exchange or removal because of any cause, which did not include adjustments to catheter positioning or anticoagulant use (e.g., heparin or urokinase) unless the catheter was removed at that time.

The secondary outcomes were the rates of complications and mortality. Complications were evaluated based on version 5.0 of the Common Terminology Criteria for Adverse Events (CTCAE), and severe complications were defined as grade ≥ 3 complications. Overall survival was calculated from the initial BAT operation or tcCVC placement to death because of any cause. In the BAT group, surgical success was defined as successful puncture for maintenance HD after the BAT procedure.

### Statistical analysis

Variables were reported as mean ± standard deviation, median (range), or number (percentage), as appropriate. Categorical variables were analyzed using the χ^2^ test, and continuous variables were analyzed using the t-test. The Kaplan–Meier method and log-rank test were used to evaluate rates of patency and overall survival. Cox proportional hazard models were used to evaluate whether the patients’ baseline characteristics were associated with the outcomes and the results were reported as hazard ratios (HRs) and 95% confidence intervals (CIs). Differences were considered statistically significant at p-values of < 0.05. All analyses were performed using EZR (Saitama Medical Center, Jichi Medical University, Saitama, Japan), which is a graphical user interface for R software (The R Foundation for Statistical Computing, Vienna, Austria)^46^.

### Ethical approval

The retrospective study protocol was approved by the ethics committee of Shizuoka General Hospital (SGHIRB#202089).

## Supplementary Information

Supplementary Information.

## Data Availability

Patient consent was not obtained for disclosure of the raw data, which are not freely available. However, an anonymized dataset can be requested from the Clinical Trials Management Office (Shizuoka General Hospital, 4-27-1 Kita-Ando, Aoi-ku, Shizuoka, 420-8527 Japan, E-mail: chiken-sougou@shizuoka-pho.jp. All requests must be approved by the Steering Committee and must be accompanied by data analysis plan.
