# Supplementary material for: Brachial artery transposition versus catheters as tertiary vascular access for maintenance hemodialysis: a single-center retrospective study
Source: Sci Rep. 2022 Jan 10;12:306. doi: 10.1038/s41598-021-03860-1 (PMC8748867; doi:10.1038/s41598-021-03860-1)
Supplement: Supplementary file 1 — Supplementary Information. [file 41598_2021_3860_MOESM1_ESM.docx]

**Supplementary Information**

**Brachial artery transposition versus catheters as tertiary vascular access for maintenance hemodialysis: a single-center retrospective study**

Yu Soma1, Masaaki Murakami*1, Eiji Nakatani2, Yoko Sato2, Satoshi Tanaka1, Kiyoshi Mori1, Akira Sugawara1

**Supplementary Table 1.** Detailed cause of CHF and hand ischemia

|  | Cause | BAT | tcCVC |
| --- | --- | --- | --- |
|  |  | n | n |
| CHF | Total | 13 | 8 |
|  | HFrEF only * | 3 | 3 |
|  | IHD only | 0 | 0 |
|  | HVD only | 1 | 1 |
|  | HFrEF and IHD | 5 | 3 |
|  | IHD and HVD | 0 | 0 |
|  | HVD and HFrEF | 4 | 1 |
|  | HFrEF and IHD and HVD | 0 | 0 |
| Hand ischemia | Total | 10 | 5 |
|  | Stage 1 † | 3 | 3 |
|  | Stage 2 | 6 | 2 |
|  | Stage 3 | 0 | 0 |
|  | Stage 4 | 1 | 0 |

BAT: brachial artery transposition, tcCVC: tunneled cuffed central venous catheter, CHF: chronic heart failure, HFrEF: heart failure with reduced ejection fraction, IHD: ischemic heart disease, HVD: heart valve disease

* In the BAT group, 3 patients had only HFrEF that was caused by dilated cardiomyopathy (DCM) in all patients. In the tcCVC group, 3 patients had only HFrEF that was caused by DCM (1 patient) or unknown causes (2 patients).

† In the BAT group, 3 patients had VA-induced stage 1 ischemia, including 2 patients who selected BAT because of the AVG infection and 1 patient who selected BAT because of radial artery occlusion. In the tcCVC group, 3 patients had VA-induced stage 1 hand ischemia stage 1, including 2 patients who selected tcCVC because of frequent AVG occlusion, and 1 patient who selected the tcCVC because of AVG infection.

**Supplementary Table 2.** New or additional VA after loss of patency for the whole access circuit

| Type of new VA | BAT (n=3) | tcCVC (n=8) |
| --- | --- | --- |
|  | n | n |
| BAT | 0 | 3 |
| Femoral artery transposition | 1 * | 0 |
| tcCVC | 2 | 3 |
| AVG | 0 | 1 ‡ |
| Peritoneal dialysis * | 0 | 1 |

VA: vascular access, BAT: brachial artery transposition, tcCVC: tunneled cuffed central venous catheter.

* In the BAT group, 1 patient added femoral artery transposition.

‡ In the tcCVC group, 1 patient changed to AVG because his vessels’ condition had improved at the time of the re-evaluation.

**Supplementary Table 3.** Predictors of mortality in the BAT and tcCVC groups

| Variable (reference) | Category | HR (95% CI) | p-value |
| --- | --- | --- | --- |
| Age | 1 | 1.03 (0.99–1.07) | 0.156 |
| Sex (male) | Female | 0.86 (0.43–1.74) | 0.676 |
| BMI | 1 | 0.85 (0.75–0.97) | 0.013 |
| Diabetes (absent) | Present | 1.08 (0.54–2.15) | 0.828 |
| Hypertension (absent) | Present | 0.51 (0.21–1.22) | 0.132 |
| IHD (absent) | Present | 1.61 (0.82–3.18) | 0.166 |
| PAD (absent) | Present | 1.79 (0.82–3.89) | 0.143 |
| Stroke (absent) | Present | 0.80 (0.36–1.78) | 0.587 |
| HFrEF (absent) | Present | 0.69 (0.35–1.38) | 0.295 |
| HVD (absent) | Present | 0.63 (0.26–1.53) | 0.320 |
| COPD (absent) | Present | 1.26 (0.54–2.92) | 0.597 |
| Cancer (absent) | Present | 1.36 (0.59–3.14) | 0.477 |
| Antiplatelet drugs (absent) | Present | 1.33 (0.67–2.62) | 0.414 |
| Warfarin (absent) | Present | 0.68 (0.28–1.67) | 0.402 |
| Disease (diabetes) | BNS | 0.44 (0.14–1.37) | 0.157 |
|  | CGN | 0.46 (0.16–1.33) | 0.153 |
|  | Others | 0.90 (0.40–2.04) | 0.805 |
| Indication (CHF) | AV access | 3.19 (1.14–8.97) | 0.028 |
|  | CVO | 1.18 (0.34–4.06) | 0.798 |
|  | Inadequate vessels | 3.35 (1.28–8.75) | 0.014 |
|  | Limited life expectancy | 2.58 (0.52–12.78) | 0.247 |
| Original type of VA (none) | AVF | 0.86 (0.38–1.97) | 0.724 |
|  | AVG | 0.94 (0.39–2.27) | 0.899 |
| Dialysis history | 1 | 1.00 (0.95–1.05) | 0.929 |
| Kind of VA (tcCVC) | BAT | 0.58 (0.28–1.22) | 0.151 |

HR: hazard ratio, CI: confidence interval, BMI: body mass index, IHD: ischemic heart disease, PAD: peripheral artery disease, HFrEF: heart failure with reduced ejection fraction, HVD: heart valve disease, COPD: chronic obstructive pulmonary disease, BNS: benign nephrosclerosis, CGN: chronic glomerulonephritis, CHF: chronic heart failure, AV access: arteriovenous access, CVS/O: central venous stenosis or occlusion, VA: vascular access, BAT: brachial artery transposition, tcCVC: tunneled cuffed central venous catheter.

Supplementary Appendix 1 Causes of death

Nineteen patients died in the BAT group. The causes of death were acute coronary syndrome (2 patients), malignancy (2 patients), sepsis unrelated to vascular access (VA, 5 patients), cirrhosis (1 patient), gastrointestinal perforation (1 patient), CO_2_ narcosis due to chronic obstructive pulmonary disease (1 patient), hemorrhage unrelated to the VA (1 patient), and unknown causes (4 patients).

Thirteen patients died in the tcCVC group. The causes of death were sepsis unrelated to VA (6 patients), malignancy (3 patients), hemorrhagic duodenal ulcer (2 patients), heart failure (1 patient), and unknown causes (2 patients).
